# Isolated mucosal leishmaniasis: A rare presentation from an endemic region of Nepal

**DOI:** 10.1016/j.jdcr.2026.06.042

**Published:** 2026-06-26

**Authors:** Asmita Gautam, Sunil Jaiswal, Badri Chapagai, Randhir Kumar Jha, Anisha Uprety, Intjar Khan, Gunja Jaiswal

**Affiliations:** a Department of Dermatology, Bheri Hospital, Nepalgunj, Nepal; b Department of Dermatology, Chitwan Medical College, Bharatpur, Nepal; c Department of Pathology, Sushil Koirala Prakhar Cancer Hospital, Khajura, Banke, Nepal; d Department of Dentistry, Chitwan Medical College, Bharatpur, Nepal

**Keywords:** leishmaniasis, miltefosine, mucosal variant, neglected tropical disease

## Introduction

Leishmaniasis is a vector-borne–neglected tropical disease caused by protozoa of the genus *Leishmania* and transmitted through the bite of infected female sandflies of the *Phlebotomus* and *Lutzomyia* genera.^1^ The estimated annual incidence of leishmaniasis is 700,000 to 1 million globally.^2^ Leishmaniasis is categorized as cutaneous leishmaniasis (CL), mucocutaneous leishmaniasis, and visceral leishmaniasis (VL) according to the clinical presentation.^3^ *Leishmania donovani*, *L braziliensis*, and *L major* are protozoans usually associated with VL, mucocutaneous leishmaniasis, and CL, respectively.^4^

Isolated mucosal leishmaniasis (ML) is a rare form of leishmaniasis even in endemic regions.^5^ Patients present with granulomatous nodules, polypoid, or exophytic mass involving the buccal mucosa, tongue, lips, palate, pharynx, larynx, and nose.^6^ Early identification and treatment of ML is essential because untreated ML may lead to nasal septum perforation and destruction, facial deformities, airway obstruction, and death.^5^

Isolated ML without a history of cutaneous or visceral involvement is uncommon.^7^ We hereby present a rare case of an isolated ML involving the oral mucosa without prior CL or VL.

## Case presentation

A 28-year-old man, a resident of Surkhet, presented with a history of painful lesions in the oral cavity for 1 year. Initially, an erosion developed on the lower lip of the patient, which gradually extended to the upper lip followed by progressive involvement of the hard palate, soft palate, tongue, and gingiva (Fig 1, *A-D*). The patient experienced a burning sensation while consuming spicy foods, which later progressed to difficulty in swallowing solid food. Over time, he also experienced difficulty in speech. There was no history of fever or constitutional symptoms. The patient denied a history of skin lesions and significant medical history other than hemorrhoids. There was no history of tuberculosis, diabetes, chronic systemic illness, or immunosuppression. A detailed history did not reveal travel to areas endemic for *L braziliensis*. There was no relevant family history or known clustering of cases.

**Fig 1:**
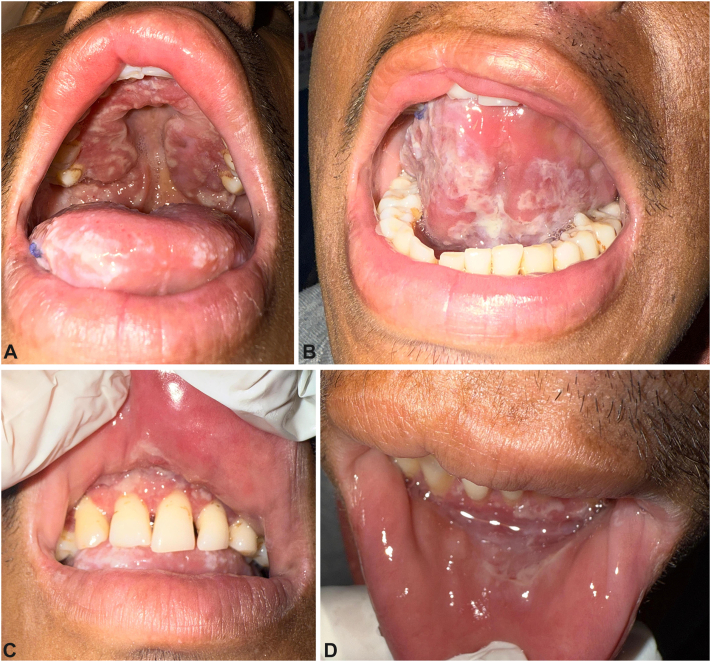
**A-D,** Exophytic growth involving the hard and soft palate with erosion present on the inner aspect of the upper and lower lip, tongue, and gingiva.

Approximately 6 months after presentation, he visited our center seeking consultation. Examination revealed erosions over the inner mucosal surfaces of both upper and lower lips, and an indurated, exophytic growth involving the hard and soft palates around the perialveolar region. Multiple ulcers were present on the bilateral lateral borders, ventral and dorsal surface of the tongue, and gingiva. Multiple cervical lymph nodes were palpable.

A computed tomography scan of the neck showed diffuse thickening of the nasopharynx, oropharynx, and laryngopharynx, along with involvement of the soft and hard palates, and multiple cervical and paraaortic lymphadenopathies. An initial biopsy taken from the lateral border of the tongue revealed a single nest of dyskeratotic squamous cells surrounded by an ulcerative area and rare atypical mitosis suggestive of malignancy. Serology for leishmaniasis (ie, rk39) was negative. A repeat biopsy from the perialveolar growth demonstrated numerous intracellular and extracellular leishmanial amastigotes bodies with an ill-defined granuloma, confirming the diagnosis of ML (Fig 2, *A-C*). CL was excluded based on the absence of current or healed cutaneous lesions on detailed dermatologic examination. Similarly, VL was ruled out by the absence of systemic symptoms (prolonged fever and weight loss), pancytopenia, negative rk39, and hepatosplenomegaly on computed tomography abdomen, supporting the diagnosis of isolated ML. The patient was subsequently started on miltefosine 50 mg twice daily, with significant clinical improvement noted within 1 month of therapy (Fig 3, *A-C*).

**Fig 2:**
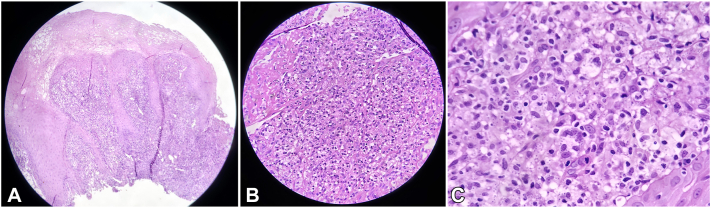
**A-C,** Histopathology shows numerous Leishman-Donovan bodies with an ill-defined granuloma. (Original magnifications: **A,** ×10; **B,** ×20; **C,** ×40.)

**Fig 3:**
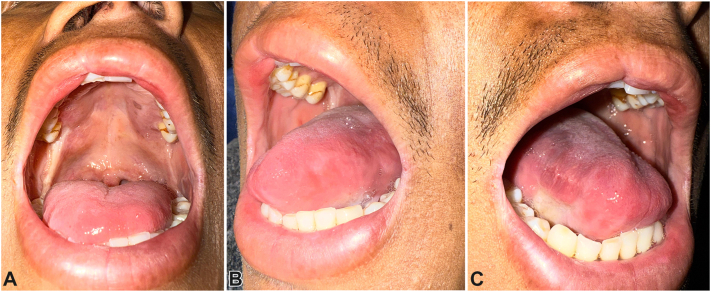
**A-C,** Resolution of oral lesions after 1 month of treatment with oral miltefosine.

## Discussion

Our study emphasizes the presentation of isolated ML involving the oral cavity, without a history of CL, in a patient from an endemic zone of Nepal.

Globally, leishmaniasis has been reported in 88 countries, mainly in South and Central America, Africa, Asia, and southern Europe.^8^ VL is endemic in 18 districts of Nepal; however, cases have been reported from 50 nonendemic districts.^9^ Initially, VL was highly prevalent in the adjacent districts of the southern and eastern Terai region adjoining the state of Bihar in India. The geographical distribution of the disease has shifted and is endemic in hilly and mountain regions as well (Fig 4).^10^

**Fig 4:**
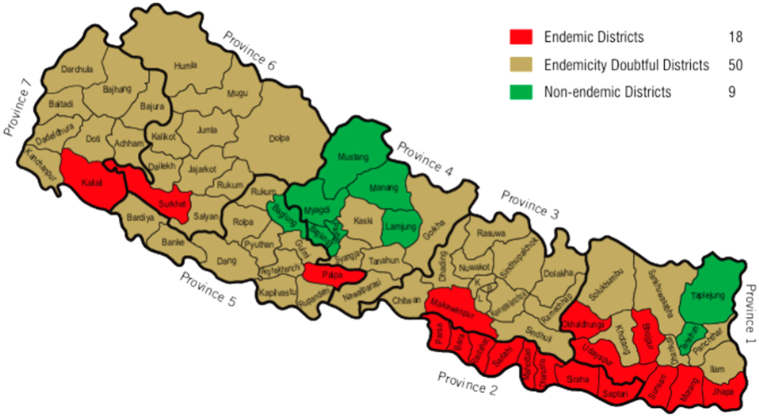
Geographical map of Nepal showing endemic districts of visceral leishmaniasis according to the National Guideline on Kala-azar Elimination Program 2019.

Nepal is endemic for VL; however, mucosal variants are rare.^7^ ML cases are predominantly confined to South America, especially in Brazil, Bolivia, Ethiopia, and Peru. In South America (New World), ML is often caused by *L braziliensis* and less commonly by *L guyanensis*, *L panamensis*, or *L amazonensis*, whereas *L infantum* has been associated with ML in the Old World.^3^^,^^5^^,^^11^^,^^12^ The common organism causing ML in Asia is *L donovani*.^2^ Travel to endemic areas, migration, poverty, malnutrition, poor hygiene, immunocompromised state, and environmental factors are the predisposing factors to develop ML.^13^ Exaggerated T helper 1 immune response in ML produces more IFN-γ and tumor necrosis factor-α, leading to tissue damage. Patients with ML have a decreased ability to down modulate the immune response by interleukin 10.^14^ Isolated ML is a rare manifestation reported in immunocompetent individuals.^11^^,^^15^

Although mucosal involvement usually develops simultaneously or months to decades after cutaneous involvement, not all patients with ML report a history of CL or VL, as observed in our patient.^4^ Oral and nasal mucosa are commonly affected, and patients present with nodules, ulcers, and nonhealing exophytic growths, as in our case, causing destruction and disfigurement.^2^^,^^3^ Infection from the oral cavity can further spread to the oropharynx and larynx, affecting the cartilage and vocal cords.^3^ Oral involvement can lead to dysphagia and difficulty in mastication, and nasal obstruction, hoarseness of voice, epistaxis, or difficulty in breathing can be associated with endonasal leishmaniasis.^15^^,^^16^

Diagnosis of ML is often challenging because it can mimic blastomycosis, syphilis, tuberculosis, leprosy, sarcoidosis, midline granuloma, Wegener disease, and neoplasms.^15^ The diagnosis of ML can be confirmed by demonstration of leishmanial amastigotes in Giemsa–stained histopathologic sections, immunohistochemistry, and detection of leishmanial DNA via polymerase chain reaction. Leishmanial amastigotes are aflagellated, small-sized, oval-to-round intracellular or extracellular structures with a nucleus and a kinetoplast.^17^ Although polymerase chain reaction has high sensitivity and specificity, it is not readily available in our setting. Serology might be negative in ML due to low titer of circulating antibodies against *Leishmania*.^6^

The treatment of choice for ML is pentavalent antimonials (meglumine antimoniate or sodium stibogluconate) in Latin America. Oral miltefosine received the Food and Drug Administration approval as an alternative treatment for ML in 2014. Pentavalent antimonials were not available in our setting. Studies show that healing of mucosal lesions is comparatively faster, and adverse effects are mild and tolerable in patients treated with miltefosine as compared with pentavalent antimonials.^18^^,^^19^ Thus, we chose oral miltefosine for our patient. Patients refractory to these agents are treated with Amphotericin B.^18^

Species identification using polymerase chain reaction could not be performed, which represents a limitation of the present study. Molecular characterization would have enabled precise species identification and strengthened the epidemiological significance of the case. Previous studies from Nepal have predominantly reported *L donovani* as the common causative agent; however, data regarding species associated with mucosal involvement remain limited.^20^^,^^21^ Further studies incorporating molecular techniques are warranted to better characterize the clinicopathological spectrum of ML in this region.

## Conclusion

Isolated ML is a rare but an important clinical entity that occurs without prior cutaneous involvement. The diagnosis of isolated ML can be difficult and often requires a high index of clinical suspicion, especially in endemic areas. Prompt recognition and appropriate systemic treatment can prevent significant disfigurement and fatal consequences, ensuring favorable outcomes.

## Conflicts of interest

None disclosed.
